# Alcohol and the Public Health

**Published:** 1915-01

**Authors:** J. N. Hurty

**Affiliations:** State Health Commissioner of Indiana


### Alcohol and the Public Health.

J. N. HURTY, M. D., STATE HEALTH COMMISSIONER OF INDIANA.

This paper shall be brief; extra brief. I am determined that that merit shall be accredited. I shall not take up the physiological action of alcohol, for that would be a work of supererogation in this presence. I shall not consider the dietetics of alcohol, for it is not a food in any sense, and has no true dietetic standing. I shall not consider the stimulant action of alcohol, for it is not a stimulant; it is only a sedative. I shall not consider the therapeutics of alcohol, for it has none. I shall not consider the mechanical or industrial uses of alcohol, for they do not here concern us.

### BUSINESS MEN.

The most important business before the business men today is the business of the public health. And, further, if the business men do not very soon grasp this truth and act upon it, then our business men are not real business men, but business children. This is said of business men because they are in the saddle, and they virtually govern and run things; and they are doing a poor job. This is attested by our over-high taxes, the failure of municipal government, the rottenness of Legislatures, the non-control of venery, the omnipresence and awful destruction of syphilis, the prevalence of preventable diseases, the prevalence and non-reduction of crime, the prevalence and non-reduction of insanity, poverty and feeble-mindedness, the increase of defectiveness and delinquency, and the increasing consumption of alcohol as a beverage with its endless chain of abominations. All of these evils, and more, are upon us, not because we cannot remove and prevent them, but because we will not. The business men, who are our leaders and governors, are continually trying to improve and increase business, succeeding only partially, because they do not recognize that business, like all other fundamentally good things of human life, depends on the moral and intellectual health of individuals. A community, a State, an empire depends upon its healthy men and women, absolutely, for its morals, strength and character. The blind, the halt, the sick, the diseased, the drug habitues and the other defectives, have no part in the prosperity and happiness of a nation. They are a source of expense and weakness. They are a burden. They must be supported, and, practically, they are useless, deleterious and unnecessary.

Assuming these statements to be true, then it follows—if a nation desires success, and is to be successful, its business men must look to it that government is righteous; and, to be this, it must closely follow the laws, so far as they are known of national well being. After governmental organization, the first absolutely necessary consideration is the cause of the public health; for without health, mental and physical, efficiency with honesty cannot exist. "The care of the public health is the first duty of the statesman," said the eminently practical Disraelli.

### HEALTH PARAMOUNT.

The public health is paramount. Do little or nothing to advance it, and the nation fails. The wonderful, well demonstrated physical and mental strength and efficiency of the Germans rests upon health. Of course, that nation has not removed all of the obstructions to health and efficiency, but it has removed many, perhaps most of the prominent ones. Among the huge obstructions to health and efficiency, yes, to life, liberty and the pursuit of happiness, is alcohol. Because of its wide use, it has become the most awful of all the drugs or dopes which have fastened upon mankind. There is no health in alcohol. On the contrary, it is an agent of physical and moral ill health.

Where squalor, immorality, bestiality and poverty exist, there alcohol and other drugs have sway. Insanity and crime trail after alcohol, and, in its wake, come ill health and disease. A high authority says: Twenty-five per cent of insanity is due to syphilis; 10 per cent is due to accident; 40 per cent is hereditary, and 25 per cent is due to alcohol. Whether or not these figures are accurate does not here greatly matter, for certain it is—alcohol is potent in the causation of insanity. It is also potent in the causation of crime, in the causation of poverty and of feeble-mindedness. Alcoholism brings sickness, and sickness induces alcoholism. Farmers are great buyers of patent medicine. Farmers who take these quack remedies generally have dyspepsia first. The dyspepsia comes from bad blood, bad cooking, and rotten teeth preventing proper mastication. When dyspepsia accepts the cordial invitation extended to it, and arrives with both feet, the victim rushes to patent medicines and finds fatuous relief in the alcohol they contain. Then diseases appear; for on top of the dyspepsia climbs alcoholism, and on it rides all kinds of neuroses.

Alcohol is clearly opposed to the public health, for it hurts any animal organism into which it is taken. It is not a food, not firstly, not secondly, not thirdly. It in no degree and in no manner aids digestion. It in no degree furthers the good of the body. On the contrary, as said, it hurts. It always hurts.

Its use as a beverage not only opposes personal health, but also the public physical and moral health, and also the public economic health. Every saloon is a public dope shop, not second in evil to the opium joint. Alcohol is truly a dope. For every dollar of revenue derived from alcoholic beverages, two dollars of public cost for crime, insanity and delinquency is endured.

### DUTY OF MEDICAL SCIENCE.

Now, what is the duty and the work of medical science in regard to alcohol? More than 260 years ago the great philosopher,

Descart, said: "If ever the human race is raised to its highest practicable level, intellectually, morally and physically, the science of medicine will perform the service." Descart was not a physician, and scholars agree he was the most original mind of this latter age, and that he more than any other thinker has moulded and directed modern scientific and speculative thought. Accepting this dictum, I ask again—what is the duty and work of medical science in regard to alcohol? From our premise, if it is accepted, that alcohol should be absolutely condemned as a beverage, and used sparingly, even reluctantly as a medicine, it is plain that the doctor, as the representative of medical science, has a fearful duty to perform.

Let the doctor then be up and doing. Let every doctor lift where he stands. Let him not dare to shirk his duty. If alcohol is not killed by the science of medicine, it will continue its destructive course, for there is no other Hercules to dispatch it.

### BUSINESS OPPOSES ALCOHOL.

After repeated appeals from the medical and surgical staff of a great manufacturing company, the controlling powers posted this notice: "Workmen frequenting drinking places, coming to or going from work, will be replaced by non-drinking men as rapidly as possible." This notice posted in the American Car and Foundry Company's plant at Berwick, Pa., has resulted in a marked decrease in accidents among 5000 men employed there. The actual decrease in accidents being 34 per. cent. There has also been a marked decrease in sickness and disease among the men and their families, and the savings banks deposits have increased $80,000 in one year.

After considering the teachings of medicine in regard to alcohol, and doubtless considering their own observations, the high military authorities of all the great nations now at war have forbidden the drinking of alcoholic liquors by their soldiers, declaring this is done in the interest of health and efficiency.

It has been suggested by the editor of a great magazine that, possibly the now raging European war is, in some degree, a war against intemperance. "After all," he argues, "our development is directed by a force or forces not ultimately under our control and higher and away from our desires and efforts." How wonderful, how passing strange it would be, if the perspective of time would disclose that a war has resulted in making man more obedient to the law of his well-being; of bringing him into closer harmony with his environment.

As a thorn in the flesh deprives one of peace and happiness, and. unless removed, will bring death, so will the thorn called alcohol, which now destroys so much flesh, soul, peace and happiness among mankind, bring more decay and final death unless it is removed.

Richard Harding Davis in a very recent newspaper article, commenting upon the troops of the allies, compares the physical condition of the Turcos with that of the whites. He says they are magnificent specimens of physical manhood; their bodies seem to be reinforced with the finest steel. They do not get sick, their endurance is twice that of the Europeans, they have not poisoned their body cells with meat and alcohol.

AN INDICTMENT.

My life experiences have forced upon me the convictions I have expressed. Alcohol is truly a greater enemy to mankind than any other drug, and we suffer incalculably from its poison, not because we cannot get from under it, but because we will not. However, I would be a rank pessimist and unworthy if I did not believe sufficiently in mankind to strongly hope that the time is not far distant when we will know the unreason of alcohol and then put it away.

In conclusion, permit me to quote a remarkable indictment against the alcoholic traffic uttered by ex-Governor Frank Hanly, of Indiana. He said:

"I bear no malice toward those engaged in the business, but I hate the traffic. I hate its every phase. I hate it for its intolerance. I hate it for its arrogance. I hate it for its hypocrisy. I hate it for its cant and craft and false pretenses. I hate it for its commercialism. I hate it for its greed and avarice. I hate it for its sordid love of gain at any price. I hate it for its domination in politics. I hate it for its corrupting influence in civic affairs. I hate it for its incessant effort to debauch the suffrage of the country; for the cowards it makes of public men. I hate it for its utter disregard for law. I hate it for its ruthless trampling of the solemn compact of State Constitutions. I hate it for the load it straps to labor's back; for the palsied hands it gives to toil; for its wounds to genius; for the tragedies of its might-have beens. I hate it for the human wrecks it has caused. I hate it for the almshouses it peoples; for the prisons it fills; for the insanity it begets; for its countless graves in potter's fields. I hate it for the mental ruin it imposes upon its victims; for its spiritual blight; for its moral degradation. I hate it for the crimes it has committed. I hate it for the homes it has destroyed. I hate it for the hearts it has broken. I hate it for the malice it has planted in the hearts of men—for its poison, for its bitterness—for the dead sea fruit with which it starves their souls.

"I hate it for the grief it causes womanhood—the scalding tears, the hopes deferred, the strangled aspirations, its burdens of want and care.

"I hate it for its heartless cruelty to the aged, the infirm and the helpless, for the shadow it throws upon the lives of children, for its monstrous injustice to blameless little ones.

"I hate it as virtue hates vice, as truth hates error, as righteousness hates sin, as justice hates wrong, as liberty hates tyranny, as freedom hates oppression."

